# Tolerability of Nasal Delivery of Humidified and Warmed Air at Different Temperatures: A Randomised Double-Blind Pilot Study

**DOI:** 10.1155/2016/7951272

**Published:** 2016-04-03

**Authors:** Susan Bibby, Sumeet Reddy, Terrianne Cripps, Steve McKinstry, Mark Weatherall, Richard Beasley, Janine Pilcher

**Affiliations:** ^1^Medical Research Institute of New Zealand, Wellington 6021, New Zealand; ^2^Capital and Coast District Health Board, Wellington 6021, New Zealand; ^3^Wellington School of Medicine & Health Sciences, University of Otago, Wellington 6021, New Zealand; ^4^Victoria University of Wellington, Wellington 6140, New Zealand

## Abstract

*Objectives*. Delivery of warmed, humidified air via nasal high flow therapy could potentially reduce replication of temperature-sensitive viruses in the upper respiratory tract. This study investigates whether nasal high flow therapy is well tolerated by healthy adults at 37°C and 41°C.* Methods*. In this randomised, double-blind, controlled crossover pilot trial, nasal high flow therapy was used to deliver humidified air at 35 L/min, at either 37°C or 41°C, for three one-hour sessions of use over one day. The alternative was delivered at least 14 days later. Ten healthy, nonsmoking adults were asked, via questionnaire after each day's use, whether they would use nasal high flow therapy while being unwell with a cold or flu if it was demonstrated to improve symptoms.* Results*. All participants completed both interventions. Eighty percent responded “yes” to future use of nasal high flow therapy, for both 37°C and 41°C. There was no significant change from baseline in saccharin times following either intervention or in the following morning.* Conclusions*. Delivering humidified air via nasal high flow therapy at both 37°C and 41°C is well tolerated by healthy adults. This supports investigation into the potential use of nasal high flow therapy as treatment in viral upper respiratory tract infections.* Trial Registration*. This trial is registered with ACTRN12614000183684 (tolerability study of nasal delivery of humidified & warmed air).

## 1. Introduction

The common cold, an acute viral infection of the upper airway, is the most frequent infection in humans [1]. It results in significant individual and public health burden due to medical visits and inappropriate prescribing of antibiotics, and it has an estimated loss of productivity of approximately 25 billion US dollars annually in the United States alone [1–3]. Although over 500 patents for common cold cures have been filed, including a wide variety of medications, natural therapies, supplements, and devices, there is no known effective treatment for the common cold [3]. One potential therapeutic measure is nasal steam inhalation, traditionally thought to offer relief from the symptoms of respiratory illnesses [4]. The pathophysiological basis of this approach is the temperature sensitivity of human rhinovirus, the causative pathogen in the majority of common cold cases [5]. Studies show that human rhinovirus may replicate optimally at 33°C, similar to the temperature of air travelling through the upper airways, rather than 37°C [6].* In vitro* studies have demonstrated human rhinovirus inhibition at 43°C within 60 minutes [7] and at 45°C within 30 minutes [8]. Influenza is also a temperature-sensitive virus, with temperature sensitivity being one of the determinants of virulence [9–11].

A Cochrane systematic review and meta-analysis compared the use of humidified air between 40°C and 47°C to humidified or room air at 30°C or below in community acquired or inoculated colds. Five randomised controlled trials with a total of 387 participants were included. No significant difference was found in symptoms, nasal resistance, or viral clearance. This lack of overall efficacy may however relate to the difficulties of delivering heated humidified air to the upper airways in an efficient and tolerable manner: for example, the indirect mode of delivery as well as distance from the nasal cavity of the commercially available Rhinotherm (Netzer Sereni, Beer Yaakov, Israel) results in a temperature drop in the humidified air, from 43 ± 1°C at the device to 39 ± 1°C at the nasal cavity [12].

Nasal high flow therapy is widely used in the inpatient and outpatient setting as a comfortable method for delivering warm, humidified room air or oxygen via nasal cannulae [13, 14]. Standard settings of 37°C are commonly used in adult patients; however a newly developed nasal high flow device (Airvo 2, Fisher & Paykel Healthcare, NZ) can deliver air or oxygen at 41°C. Unlike previous devices [7, 12, 15–17], the nasal high flow device uses a closed-loop control system to accurately deliver gas at a set dew point temperature directly to the nasal cavity.

We hypothesised that nasal high flow therapy at 41°C would be as well tolerated as the standard setting of 37°C for three sessions of one hour in one day. We used questionnaires to assess participants' responses and the saccharin test to assess nasal mucociliary function before and after nasal high flow therapy use. This pilot tolerability study was undertaken to determine whether nasal high flow therapy could be used in clinical trials to assess efficacy in reducing symptom severity and viral load in subjects suffering from the common cold or flu.

## 2. Methods

This randomised double-blind controlled crossover pilot trial was undertaken over four visits to the Medical Research Institute of New Zealand at Wellington Regional Hospital. The study was prospectively registered on the Australian New Zealand Clinical Trial Registry (ACTRN12614000183684), approved by the “Northern A” Health and Disability Ethics Committee (13/NTA/150) and sponsored by Fisher & Paykel Healthcare, NZ.

Study participants were healthy adults aged 18 years or older, without asthma or chronic obstructive pulmonary disease requiring medication in the last six months, without sinusitis or nasal spray use in the last seven days, and with no cigarette use within the last month. Recruitment was by local advertisement and word of mouth. Potential participants were not eligible if they had any symptoms in the last seven days consistent with an upper respiratory tract infection. These symptoms could include any of sore throat, cough, nasal discharge, or raised temperature. Potential participants were also not eligible if they had any other condition which, at the investigator's discretion, could be a safety risk for the participant or affect the interpretation of the study outcome variables, such as primary ciliary dyskinesia, cystic fibrosis and nasal septal deviation, or other abnormalities of the nose.

At the first visit written informed consent was obtained and baseline demographic information was collected. A saccharin test was administered, reported here as the “baseline” measurement. After visual inspection to exclude any obvious anatomical abnormalities, fifty microlitres of saccharin solution was placed on the surface of the inferior turbinate of the right nostril, based on previous methodology [18–20]. Participants were instructed to try not to breathe through their nose, sniff, cough, eat, drink, or talk. The time from saccharin insertion to taste of saccharin was recorded. The test was stopped if the participant did not taste saccharin within 45 minutes.

The study had AB/BA crossover design. Each participant was assigned by an opaque randomisation envelope in the order in which they received the two interventions described below. The randomisation schedule was generated using a computer-based randomisation program by the study statistician (Mark Weatherall), who was not involved in trial recruitment, procedures, or data collection. All other investigators were blinded to the intervention order up to the time of randomisation. The subsequently unblinded investigator was then responsible for nasal high flow administration only. Thus the investigators responsible for saccharin testing and questionnaire administration, as well as the participants, remained blinded to the order of the treatments for the duration of the data collection.

The two interventions were delivery of heated humidified air via nasal high flow therapy (Airvo 2, Fisher & Paykel Healthcare) at either 37°C (“NHF 37C”), that is, standard temperature setting, or 41°C (“NHF 41C”), using a nasal high flow device modified for this purpose by the manufacturer. Air was delivered via nasal cannulae (Optiflow size Medium, Fisher & Paykel Healthcare) at 35 litres per minute and the machine was allowed at least 30 minutes to warm up to ensure that the temperature had reached the correct set point. Three 60-minute sessions were delivered, separated by at least two hours. Participants were able to stop the intervention at any time without being withdrawn from subsequent portions of the study. They were also able to withdraw from the study at any time.

After the third session of each intervention the Intervention Tolerability Questionnaire was administered (see below). At least 10 minutes after completion of the third session of each intervention, another saccharin test was performed (“same day” measurement). The following day the participant returned for another saccharin test (“next day” measurement), between 22 and 26 hours after the start of the first nasal high flow therapy session.

**Figure 1:**
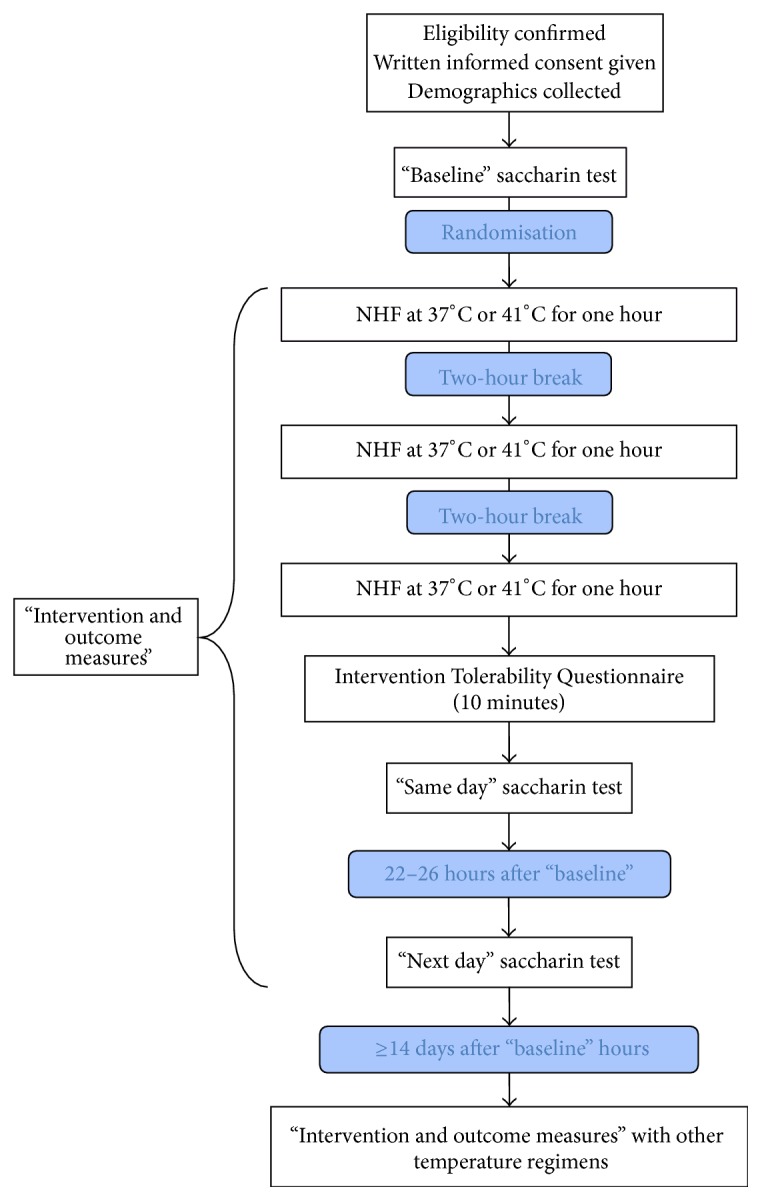
Study design.

At least 14 days following the first intervention, participants attended again for their randomised crossover treatment with the same sequence of measurements (Figure 1).

The primary outcome was the questionnaire response to “If the Airvo was shown to improve cold and/or flu symptoms would you be willing to use the Airvo for 1 hour three times a day while unwell?” If the participant answered yes, they then answered “Would you be willing to wear the Airvo for longer periods of time?” and “Would you be willing to try wearing the Airvo overnight?” If the participant answered no, they then answered “Please explain the characteristics of the Airvo which would mean you would not be willing to wear it” and “What is the maximum length of time (if any) in a 24 hour period would you be prepared to wear the Airvo for while unwell?”

Secondary outcomes included five-level Likert scaled questions rating general comfort, comfort of nasal passages, weight of nasal interface, noise of the intervention, and ease of breathing through nose. On the scale, one reflected the most favourable response and five reflected the least favourable. Other outcomes were the proportion of participants who tolerated each session for 60 minutes, the length of time a session was tolerated for if less than 60 minutes, mean saccharin test times, and the number of participants who had a normal initial baseline saccharin test that became abnormal after the intervention. An abnormal test was conservatively defined as >20 minutes until taste [21, 22].

Simple data descriptions are shown. For continuous variables a mixed linear model was used, with the order of treatment and baseline reading as fixed effects covariates and the individual participants as a random effect to take into account the crossover design. For categorical variables McNemar's exact test for equality of paired marginal proportions was used for two-by-two tables. Confidence intervals for the paired comparisons of the proportions in the “NHF 41C” data and the proportions in the “NHF 37C” data were also shown but do not correspond to *P* value as they are estimated using asymptotic methods. The ordinal variables were summarised and the Likert scale questions were presented by dichotomising at the boundary point of 3+ higher scores. A power calculation was not used for this pilot tolerability study.

SAS version 9.3 was used.

## 3. Results

**Figure 2:**
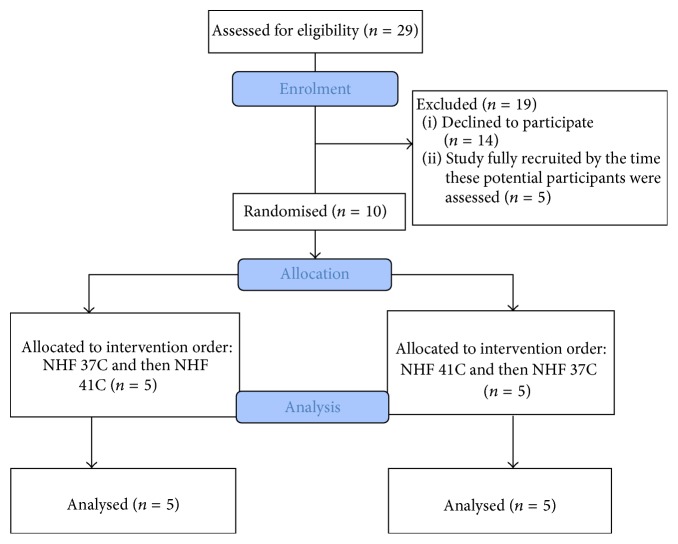
Flow of participants through the study.

Between March and May 2014, 29 potential participants were assessed for eligibility (Figure 2) and ten were randomised. Participant characteristics are shown in Table 1. None of the participants had any current respiratory diagnoses: one reported allergic asthma as a child; another used a nasal spray with colds but had not taken it for at least a month; one further reported hypertension; and another had undergone an abdominal aortic aneurysm repair some years earlier. All were New Zealand European, though this ethnic group was not actively sought for inclusion in the study.

### 3.1. Nasal High Flow Therapy Questionnaire Results

Every participant completed all of the sessions and measurements for both interventions. The median (range) time between interventions was 14 (14 to 104) days. Participants were asked whether they would be prepared to use nasal high flow therapy three times a day while being unwell, if it was demonstrated to improve cold and/or flu symptoms. Eight out of 10 participants said “yes” for “NHF 37C,” and the same eight participants said “yes” for “NHF 41C.” Two participants were not prepared to wear either “NHF 37C” or “NHF 41C” for colds or influenza and felt that shorter time periods would not make nasal high flow therapy more tolerable to them. One participant stated that he felt it was only suited to more severe respiratory illnesses (for which he would wear nasal high flow therapy for “24 hours a day” if necessary) while the other felt the physical restriction of wearing nasal high flow therapy would make her prefer alternative remedies such as medication and rest. Of the eight participants that would use nasal high flow therapy, five stated they would be prepared to wear “NHF 37C” for longer periods, and three of these stated they would be prepared to wear “NHF 41C” for longer periods. The proportion of participants willing to try wearing “NHF 37C” and “NHF 41C” overnight were four of the eight participants for both devices.

Table 2 shows the comparisons for the Likert scaled questions between the two treatments. Free text comments had several recurring themes and these included decreased mobility, damp/dripping nose, the noise of the machine, difficulty in breathing, and sneezing. Other comments were “(I would use it) for as long (and) as often as needed,” “this system is superb,” and “(it would be) difficult to use if (I) had a blocked nose.”

### 3.2. Saccharin Test Results

Saccharin test times are shown in Table 3. There was no statistically significant difference (*P* > 0.05) between the saccharin times following the different interventions. The estimate (95% CI) of the difference between “NHF 41C” and “NHF 37C,” with order of randomisation and baseline measurement as covariates, on “same day” was 1.0 (−4.15 to 2.13) minutes, *P* = 0.51, with the “next day” estimate of 9 (−1.12 to 19.88) minutes, *P* = 0.076.

### 3.3. Adverse Events

Three participants reported a total of four mild headaches, two after “NHF 37C” and two after “NHF 41C.” Other events possibly related to nasal high flow therapy were one “runny nose” and one event of “dry eyes” following “NHF 41C,” and the sensation of “not being able to take a deep breath” during “NHF 37C” use. All of these events were classified as mild and resolved within an hour or less of finishing nasal high flow therapy.

## 4. Discussion

In this study, we show that delivery of warmed humidified air at 35 L/min at both 37°C and 41°C is well tolerated by healthy adults for periods of one hour, three times a day. Eight out of the 10 participants said they would use nasal high flow therapy for cold and/or flu symptoms according to the trial protocol, were it found to be effective. None of our participants chose to stop nasal high flow therapy in this study, although all had the option of doing so. About half of the participants would be willing to wear either device for longer than three, one-hour sessions or were willing to try it overnight.

After using nasal high flow therapy all participants reported that their nasal passages were at least moderately comfortable (scores 1–3/5) with no differences between interventions. Reported scores for general comfort, weight of nasal interface, noise of the intervention, nasal passage comfort, and ease of breathing through nose did not differ significantly between interventions. Scores were more favourable after the “NHF 37C” intervention (median differences 0.5 points to 1 point for 4 of the 5 variables), but this difference was not statistically significant and is unlikely to be important from a clinical perspective, particularly as the largest difference between the two interventions was for “noise” which did not differ between the two machines.

Interpretation of the saccharin tests was limited due to the insensitivity of this test, and the inherent within- and between-participant variability of this measurement [21, 23–25], with some values as low as 2 seconds (unlikely to be due to ciliary movement) and others not tasted after 45 minutes (the cut-off time). We were reassured that in no participants did normal “baseline” results rise to an abnormal value directly after nasal high flow therapy. Also, we note that facial burns victims have been shown to have normal saccharin times despite exposure to higher temperatures than given here [26].

Nasal high flow therapy was delivered for an hour at each session, as this was the time shown* in vitro* to inhibit human rhinovirus [7], and it was thought to be a reasonable time to expect people to wear the device in the “real world” situation. This proved to be the case, as all participants tolerated nasal high flow therapy for this regimen, the majority of participants indicated that they would be willing to trial it upon suffering from cold or flu, and about half indicated that they would use it for longer periods, including overnight. Clearly the small sample size means the study lacks statistical power to determine differences between the two interventions; however it was adequate to assess their tolerability. We chose to exclude potential participants with asthma or chronic obstructive pulmonary disease, although we recognise that assessment of tolerability in such groups will be necessary; those with preexisting respiratory illnesses are in greater need for effective therapeutic measures, with viral respiratory tract infections being an important cause of severe exacerbations of asthma and chronic obstructive pulmonary disease [27].

## 5. Conclusion

We have shown that nasal high flow therapy is well tolerated by healthy volunteers when administered at 35 L/minute and that increasing the temperature from 37°C to 41°C does not affect tolerability. This paves the way for further trials investigating the tolerability and efficacy of this higher temperature for symptom relief in respiratory virus infections such as the common cold and influenza and for reducing the severity of exacerbations of chronic respiratory illnesses.

## Figures and Tables

**Table 1:** Participant characteristics.

Categorical variables	*N* (%)	
Male	4 (40)	
Past smoker	4 (40)	

Continuous variables	Median (IQR)	Min to Max

Age (years)	53.3 (34.7 to 62.8)	26.4 to 73.1
Pack years	0 (0 to 2)	0 to 33
Body mass index (kg/m^2^)	25.1 (23.2 to 27.7)	20.7 to 34.4

*N* = 10.

**Table 2:** Tolerability Questionnaire results^*∗*^.

Individual question results									
Median (minimum to maximum)									
General comfort		Nasal interface weight		Noise		Nasal passage comfort		Ease of breathing through nose	
NHF 37C	NHF 41C	NHF 37C	NHF 41C	NHF 37C	NHF 41C	NHF 37C	NHF 41C	NHF 37C	NHF 41C

2.5 (1 to 3)	3.0 (1 to 4)	1.5 (1 to 3)	2.0 (1 to 3)	2.0 (1 to 3)	3.0 (2 to 4)	2.0 (1 to 3)	2.0 (1 to 3)	2.5 (1 to 4)	3.0 (1 to 4)

Difference in proportion that scored 3 or above (95% CI), NHF 41C minus NHF 37C^∧^									
General comfort		Nasal interface weight		Noise		Nasal passage comfort		Ease of breathing through nose	

30% (1.6 to 58.4) *P* = 0.25		0% (−39.2 to 39.2) *P* = 0.99		20% (−4.8 to 44.8) *P* = 0.50		20% (−4.8 to 44.8) *P* = 0.50		20% (−4.8 to 44.8) *P* = 0.50	

Free text comments^#^									
Intervention		Difficulty in breathing through the nose		Damp/dripping nose	Noise of machine	Decreased mobility		Sneezing	

NHF 37C		5		5	1	2		0	
NHF 41C		9		5	4	3		2	

*N* = 10. ^*∗*^On a five-point Likert scale, values close to one are favourable and values close to five are less favourable;  ^∧^ *P* values by McNemar's exact test, confidence intervals by asymptotic method. ^#^The same participant could be counted twice if they made the same comment for each intervention.

**Table 3:** Saccharin test times.

Treatment	Mean (SD) and median (minimum to maximum)		
	Baseline	Same day but after NHF	Next day, after NHF
NHF 37C	11 (16)^*∗*^	6 (4)	4 (3)
	3 (0 to 45)^*∗*^	5 (3 to 18)	3 (0 to 9)

NHF 41C	8 (13)	4 (4)	12 (17)^∧^
	5 (0 to 43)	4 (0 to 10)	5 (0 to 45)^∧^

Times are in minutes. NHF = nasal high flow.

*N* = 10, with the following exceptions:

^*∗*^For one of the participants, the baseline saccharin prior to “NHF 37C” was not tasted within 45 minutes; all of their other test times were normal.

^∧^For one of the participants, the next day saccharin after “NHF 41C” was not tasted within 45 minutes; all of their other test times were normal.

The data points for when saccharin was not tasted within 45 minutes were assigned a value of 45 minutes.

One participant had abnormal saccharin taste times (>20 minutes, but under 45 minutes) for both baseline tests and for the “day after” test following “NHF 41C” but normal results directly after both “NHF 37C” and “NHF 41C.”

The other seven participants had normal baseline, “same day,” and “next day” measurement times (≤20 minutes).
